# Neighbourhood effects on loneliness among adolescents

**DOI:** 10.1093/pubmed/fdad053

**Published:** 2023-05-12

**Authors:** Jose Marquez, Pamela Qualter, Kimberly Petersen, Neil Humphrey, Louise Black

**Affiliations:** Manchester Institute of Education, University of Manchester, Manchester M13 9PL, UK; Manchester Institute of Education, University of Manchester, Manchester M13 9PL, UK; School of Education, University of Leeds, Leeds LS2 9JT, UK; Manchester Institute of Education, University of Manchester, Manchester M13 9PL, UK; Manchester Institute of Education, University of Manchester, Manchester M13 9PL, UK

**Keywords:** adolescence, epidemiology, loneliness, neighbourhood, wellbeing

## Abstract

**Background:**

Loneliness is a growing public health concern, but little is known about how place affects loneliness, especially during adolescence. This is the first study to examine the influence of neighbourhoods on loneliness in early-to-mid adolescence.

**Methods:**

Baseline data from the #BeeWell cohort study in Greater Manchester (England), including 36 141 adolescents (aged 12–15 years) across 1590 neighbourhoods, were linked to neighbourhood characteristics using administrative data at the level of lower super output areas and analysed using multilevel regression.

**Results:**

Neighbourhood differences explained 1.18% of the variation in loneliness. Ethnic, gender and sexual orientation inequalities in loneliness varied across neighbourhoods. Several neighbourhood characteristics predicted loneliness at the individual level, including skills deprivation among children and young people, lower population density and perceptions of the local area (feeling safe; trust in local people; feeling supported by local people; seeing neighbours as helpful; the availability of good places to spend free time). Finally, a longer distance from home to school was associated with significantly higher loneliness.

**Conclusions:**

Neighbourhoods account for a small but significant proportion of the variation in adolescent loneliness, with some neighbourhood characteristics predicting loneliness at the individual level, and loneliness disparities for some groups differing across neighbourhoods.

## Introduction

Loneliness occurs when a person thinks their interpersonal relationships are insufficient in some way.^1^ It is a subjective experience, accompanied by painful or negative emotions^2^ and a perceived lack of connectedness to peers during adolescence.^3^ Although there is substantial indication that individual factors are associated with loneliness, little is known about how place affects loneliness, especially among adolescents.^4^ We therefore set out to determine whether neighbourhoods and their characteristics influence adolescent loneliness. This follows the recent Department for Culture, Media & Sport Loneliness Evidence Gap report,^5^ which called for more research on place-based factors that are likely to contribute to loneliness, given that spatial environments affect mental health, mainly via opportunities for increased social interactions.

Though not specifically developed with adolescence in mind, Lim *et al*.’s conceptual model^6^ offers a useful theoretical framework, emphasizing as it does the importance of socio-demographic and social–environmental factors as contributors to loneliness, and the interplay between micro- and macro-level influences. In a similar vein, Bronfenbrenner’s ecological systems theory (EST) highlights the neighbourhood as a micro-systemic developmental context, while also noting neighbourhood characteristics as exo-systemic factors that can influence experiences and outcomes (including loneliness) of the developing person.^7^ As with Lim’s model, EST also emphasizes the interaction between different systemic influences as critical to our understanding of a given phenomenon, such as loneliness. Drawing on these related perspectives, we position our study as one that explores the influence of a specific developmental context and its characteristics (research questions 1 and 3 below), while also examining how the interaction between individual and context can shape outcomes (research question 2 below).

Defining ‘place’ can be challenging. Consideration needs to be given to the most appropriate unit of analysis, to minimize the uncertain geographic context problem. This occurs when observed effects of neighbourhoods and their characteristics are sensitive to how they are geographically demarcated, and the extent to which this varies from the ‘true causally relevant’ geographic context.^8^ This is particularly pertinent in studies involving children and adolescents because most area effects studies rely on administrative units of analysis (e.g. census tract data^9^). In rare cases where the demarcation of place is built around how residents experience and define their neighbourhood,^10^ only the views of adults are typically sought.^11^

In the current absence of adolescent-derived neighbourhood demarcation, use of lower super output areas (LSOAs) is arguably the next best option. LSOAs are geographic units comprising~650 households (c. 1500 residents). As the most granular unit available in administrative data, they are more likely to reflect how adolescents conceptualize the geographic boundaries of their neighbourhoods than other, much larger available units such as middle super output areas, wards or Local Authority districts. This is because children and young people have less mobility and access to resources than adults and are thus more likely to spend time in their immediate local area.^12^^,^^13^ To the best of our knowledge, ours is the first study to use LSOA (or equivalently granular) geographic units in the study of loneliness among adolescents (or indeed other age groups), adding to a complex evidence base that has, as yet, failed to find consistent results regarding the causally relevant geographic context/unit or indeed the factors that might explain geographical variation in loneliness (such as relative urbanicity).^14^ These issues are illustrated in two recent studies, outlined below.

Marquez *et al*.^9^ showed that loneliness in mid-late adolescence (age 16–24 years) is influenced by a range of community factors (as well as socio-demographic, social, health and wellbeing influences). Indeed, 5–8% of the variation in loneliness was explained at the community level, and ethnic, gender and sexual orientation inequalities in loneliness differed across those communities. However, this study used geographic units (local authority districts in England) that differ widely in area and population size, ranging from 2224 (Isles of Scilly) to 1 141 816 (Birmingham), illustrating the above geographic context problem. By contrast, Matthews *et al*.’s^15^ study of loneliness among 12- and 18-year-olds found little evidence of the influence of neighbourhood characteristics (such as relative urbanicity, socio-economic status and population density) other than perceptions of collective efficacy and greater neighbourhood disorder. Because that study did not adopt a multilevel approach (i.e. adolescents nested in neighbourhoods), the authors were not able to estimate the variation in loneliness explained at the neighbourhood level. Both studies used data collected prior to the Covid-19 pandemic.

It is important to look at how place affects loneliness among younger adolescents, given the high prevalence rates among school-aged youth.^16^ Early-to-mid adolescence is a period characterized by significant developmental change,^17^ including substantial shifts in the form and functioning of interpersonal relationships.^18^ This period also confers significant vulnerability to low wellbeing.^19^ Previous research has shown that the influence of neighbourhoods on children’s wellbeing increases in early adolescence.^20^^,^^21^ If place can impact loneliness, there may be structural options for intervention. However, no previous study has examined the influence of neighbourhoods on loneliness in early-to-mid adolescence.

In the current study, we address the gaps and issues noted above, by exploring how place influences loneliness during early-to-mid adolescence in post-Covid-19 Greater Manchester (England), the fifth largest metropolitan area in Europe. We focus on neighbourhoods (as opposed to larger geographic units such as local authority districts) and study a range of characteristics measured at this level. Our research questions were:

(i) What proportion of the variation in adolescent loneliness is explained at the neighbourhood level?
(ii) Do adolescent inequalities in loneliness across age, ethnicity, sex, gender identity, sexual orientation and socio-economic status vary across neighbourhoods?
(iii) Which neighbourhood factors are associated with adolescent loneliness?

**Table 1:** Characteristics of the sample (36 141 adolescents, 1590 neighbourhoods) and descriptive information of study variables

Neighbourhood characteristics (no missing data)	Description	Value
Economy, work and employment:		
Child income deprivation	IoD 2019 Income Deprivation Affecting Children Index Score.^17^ Proportion of all children aged 0–15 living in income deprived families	0.21 (0.13)
Employment deprivation	IoD 2019 Employment Score.^17^ Proportion of the working age population involuntarily excluded from the labour market (i.e. people who would like to work but are unable to do so due to unemployment, sickness or disability, or caring responsibilities	0.14 (0.08)
Education:		
Children and young people skills deprivation	IoD 2019 Children and Young People Sub-domain Score.^17^ Key Stage 2 attainment, key Stage 4 attainment, secondary school absence, the proportion of young people not staying on in school or non-advanced education above age 16, and the proportion of young people aged under 21 not entering higher education	0.05 (0.82)
Adult skills deprivation	IoD 2019 Adult Skills Sub-domain Score.^17^ Proportion of working-age adults (women aged 25–59 and men aged 25–64) with no or low qualifications, and the proportion of the working-age population who cannot speak English or cannot speak English well	0.36 (0.13)
Health:		
Health deprivation and disability	IoD 2019 Health Deprivation and Disability Score.^17^ Years of potential life lost (death before the age of 75 from any cause), work limiting morbidity and disability (based on those receiving benefits due to inability to work through ill health), acute morbidity (level of emergency admissions to hospital, based on administrative records of inpatient admissions), and mood and anxiety disorders (levels of mental ill health in the local population, including mood (affective), neurotic, stress-related and somatoform disorders)	0.70 (0.72)
Crime:		
Crime	IoD 2019 Crime Score.^17^ Violence, burglary, theft and criminal damage	0.68 (0.73)
Barriers to housing and services:		
Geographical barriers	IoD 2019 Geographical Barriers Sub-domain Score.^17^ Road distance to a post office, a primary school, a local store or supermarket, and a GP surgery	−0.35 (0.61)
Wider barriers	IoD 2019 Wider Barriers Sub-domain Score.^17^ Household overcrowding (proportion of household that are classed as overcrowded), homelessness (rate of acceptances for housing assistance under the homelessness provisions of housing legislation) and housing affordability (inability to afford to enter owner-occupation or the private rental market)	−0.03 (2.08)
Living environment:		
Indoor environment deprivation	IoD 2019 Indoors Sub-domain Score.^17^ Housing in poor condition (proportion of social and private homes that fail to meet the Decent Homes standard) and the proportion of houses without central heating	0.26 (0.64)
Outdoor environment deprivation	IoD 2019 Outdoors Sub-domain Score.^17^ Air quality (concentration of the four pollutants nitrogen dioxide, benzene, sulphur dioxide and particulates) and road traffic accidents (proportion of reported accidents that involve death or personal injury to a pedestrian or cyclist)	−0.07 (0.47)
Demographic:		
Population density	Lower layer Super Output Area population density (number of people per square kilometre)^18^	4755 (3012)
#BeeWell—local environment variables (from 1 to 5)	Derived from the level of agreement reported by participants in the #BeeWell survey (Strongly disagree = 1; Disagree = 2; Neither agree nor disagree = 3; Agree = 4; Strongly agree = 5)	
I feel safe in the area where I live (8.57% missing)		4.00 (0.99)
People around here support each other with their wellbeing (9.54% missing)		3.62 (1.06)
You can trust people around here (9.23% missing)		3.55 (1.11)
I could ask for help or a favour from neighbours (9.24% missing)		3.64 (1.21)
There are good places to spend your free time (e.g. leisure centres, parks, shops) (9.06% missing)		3.85 (1.10)
Distance to school (no missing data)		
Distance from home to school	Distance from the postcode of residence to the postcode of the school (in kilometres)	2.34 (2.16)
Socio-demographic characteristics of the sample		
School year		
* Year 8 (age 12/13)*		19 467 (53.86)
* Year 10 (age 14/15)*		16 674 (46.14)
Ethnicity		
* White*		23 254 (64.34)
* Asian*		6592 (18.24)
* Black*		1954 (5.41)
* Other*		3890 (10.76)
* Missing*		451 (1.25)
Gender		
* Male*		14 605 (40.41)
* Female*		14 160 (39.18)
* Gender diverse*		2581 (7.14)
* Prefer not to say*		1931 (5.34)
* Missing*		2864 (7.92)
Sex at birth		
* Male*		18 301 (50.64)
* Female*		17 840 (49.36)
Sexual orientation		
* Heterosexual*		24 344 (67.36)
* Minority sexual orientation*		5094 (14.09)
* Prefer not to say*		3296 (9.12)
* Missing*		3407 (9.43)
FSMs eligibility (ever 6 FSM)	Pupils recorded on the School Census who were recorded as known to be eligible for FSM in any of the termly censuses in the previous 6 years	
* No*		26 382 (73.00)
* Yes*		9202 (25.46)
*Missing*		557 (1.54)
Loneliness outcome variables		
Loneliness (from 1 to 5; 9.01% missing data)	How often do you feel lonely (Never = 1; Hardly ever = 2; Occasionally = 3; Some of the time = 4; Often or always = 5)	20.73 (1.26)

Sample size (*n*) and percentage (%) are given for categorical variables, and mean and SD are given for continuous variables.

## Methods and data

### Participants

We used cross-sectional data from the first wave of the #BeeWell project,^22^ linked via residential postcode to neighbourhood-level administrative data^23^^,^^24^ The final sample resulting from merging these datasets comprised 36 141 adolescents aged 12–15 years from 1590 neighbourhoods in Greater Manchester, England. The characteristics of the sample are noted in Table 1. They closely mirror those of the population of young people aged 11–16 years both in Greater Manchester and in England (although, compared to national levels, the proportion of Asian youth is somewhat higher, and the proportion of White youth is somewhat smaller^25^).

**Table 2:** Correlation between loneliness, neighbourhood characteristics, #BeeWell local environment variables and distance from home to school

Variables	(1)	(2)	(3)	(4)	(5)	(6)	(7)	(8)	(9)	(10)	(11)	(12)	(13)	(14)	(15)	(16)	(17)	(18)
(1) Loneliness	1.000																	
(2) Child income deprivation	0.001	1.000																
(3) Employment deprivation	−0.002	0.900	1.000															
(4) Children and young people skills deprivation	0.008	0.823	0.788	1.000														
(5) Adult skills deprivation	−0.021	0.827	0.853	0.799	1.000													
(6) Health deprivation and disability	−0.001	0.847	0.885	0.803	0.785	1.000												
(7) Crime	−0.008	0.707	0.678	0.610	0.625	0.699	1.000											
(8) Geographical barriers	0.020	−0.339	−0.318	−0.302	−0.369	−0.347	−0.361	1.000										
(9) Wider barriers	−0.013	0.741	0.696	0.574	0.636	0.738	0.667	−0.443	1.000									
(10) Indoors environment deprivation	−0.005	0.054	−0.018	0.098	0.090	0.074	0.227	−0.461	0.267	1.000								
(11) Outdoors environment deprivation	−0.023	0.426	0.370	0.283	0.379	0.421	0.565	−0.454	0.598	0.399	1.000							
(12) Population density	−0.028	0.271	0.183	0.166	0.299	0.203	0.216	−0.533	0.443	0.362	0.422	1.000						
(13) Distance from home to school	0.011	−0.053	−0.053	−0.058	−0.075	−0.066	−0.053	0.085	−0.060	−0.049	−0.078	−0.095	1.000					
(14) I feel safe in the area where I live	−0.050	−0.531	−0.480	−0.510	−0.449	−0.482	−0.407	0.278	−0.421	−0.092	−0.267	−0.172	0.036	1.000				
(15) People around here support each other with their wellbeing	−0.067	−0.345	−0.301	−0.368	−0.275	−0.331	−0.263	0.157	−0.248	−0.069	−0.150	−0.044	0.017	0.654	1.000			
(16) You can trust people around here	−0.060	−0.475	−0.429	−0.462	−0.409	−0.441	−0.358	0.222	−0.388	−0.105	−0.240	−0.163	0.034	0.679	0.737	1.000		
(17) I could ask for help or a favour from neighbours	−0.066	−0.233	−0.209	−0.263	−0.153	−0.227	−0.172	0.072	−0.153	−0.028	−0.043	0.001	−0.003	0.445	0.537	0.530	1.000	
(18) There are good places to spend your free time	−0.050	−0.262	−0.250	−0.327	−0.266	−0.259	−0.194	0.027	−0.096	0.063	−0.015	0.052	0.009	0.457	0.501	0.481	0.386	1.000

### Measures

Descriptive information about the measures is presented in Table 1. Further details about the variables are presented below.

#### Geographic unit: LSOA

In the absence of available data on adolescent-informed neighbourhood demarcation, we used LSOA, as this is the most granular geographic unit available (and hence, more likely to mirror adolescents’ perceptions of the boundaries of their neighbourhood than less granular units such as seamless locales, middle super output areas or wards). LSOAs are geographic units of ~650 households (c. 1500 residents). There were 1682 LSOAs in the current study, a number that reduced to 1590 after excising those with <5 observations, consistent with guidance on multilevel modelling.^26^ For the same reason, in the analysis of random effects (see Analyses below), LSOAs with <10 observations were excluded, resulting in 1438 LSOAs.

#### Dependent variable: loneliness

We used the Office for National Statistics (ONS) single-item loneliness measure.^27^ Participants were asked how often they felt lonely (Never; Hardly ever; Occasionally; Some of the time; Often or Always).

#### Covariates

Data on participants’ age, sex, ethnicity, free school meal (FSM) eligibility (shared by the 10 Greater Manchester Local Authorities), gender identity and sexual orientation (from #BeeWell survey responses) were used as covariates.

#### Neighbourhood characteristics

We analysed 16 neighbourhood characteristics spanning the 8 domains described in Table 1. In six of them (Economy, work and employment; Education; Health; Crime; Barriers to housing and services; Living environment), data are from the Indices of Deprivation 2019, with higher scores indicating higher deprivation.^23^ In the Demographic domain, data on population density were collected by the ONS,^24^ with higher scores indicating higher population density. An additional domain includes five measures of adolescents’ perceptions of their local environment. These data were collected in the #BeeWell survey and aggregated to the neighbourhood (LSOA) level, with higher scores indicating more positive perceptions. Finally, we also examined one correlate measured at the individual level—the distance between the residential postcode and the school postcode—derived using information provided by the Greater Manchester Combined Authority.

### Analyses

Our analysis involved several stages. First, to provide a ‘benchmark’^26^ deviance value and to calculate the intraclass correlation coefficient (ICC) (Model 1), a null (empty) multilevel model (level 1: individual; level 2: neighbourhood) with no fixed predictors was estimated. Second, we fitted a multilevel model including the covariates measured at Level 1 (i.e. age, ethnicity, gender, sexual orientation and FSM) (Model 2) to examine socio-demographic inequalities in loneliness and to report the ICC after accounting for these. Next, to ascertain whether inequalities in loneliness between socio-demographic groups differ across neighbourhoods, we fitted a model that examined neighbourhood random effects (Model 3). In this model, all socio-demographic variables were retained in the fixed part of the model, and then each of them was incorporated one at a time in the random part. We used likelihood ratio (LR) tests to assess model fit improvement,^28^ which was taken as evidence of neighbourhood random effects.

To investigate whether neighbourhood characteristics are associated with loneliness, we fitted a set of multilevel models. First, neighbourhood-level factors grouped by domain (e.g. crime, population density) were introduced as explanatory variables in the same multilevel model (Model 4A, unadjusted). This results in one model per domain. Then, we fitted the same models while controlling for socio-demographic characteristics (Model 4B, adjusted). Second, we studied the unique associations between each neighbourhood characteristic and loneliness in multilevel models before (Model 5A, unadjusted) and after (Model 5B, adjusted) controlling for socio-demographic characteristics. Thus, each unique association model includes only one neighbourhood characteristic. The study of unique associations helps to identify associations that may not be observed in Models 4A and 4B due to collinearity issues.

All continuous variables were standardized to facilitate the interpretation of the results. Multilevel models were fitted using restricted maximum likelihood estimation. Multiple imputation was used to account for missing data. Levels of missing data are reported in Table 1. Twenty-five imputations of the data set were performed using a multivariate normal regression.^29^ Analyses were conducted in STATA 15.^30^

## Results

### Descriptive statistics

Table 1 presents the descriptive statistics of the sample.

### Multilevel models

The ICC indicated that 1.18% of the variation in loneliness was explained at the neighbourhood level (Model 1). After controlling for socio-demographic characteristics (Model 2), this reduced to 0.37%.

The results of Model 3 are presented in Table A1 in Appendix 1. Based on the LR tests, we found that neighbourhoods influenced loneliness inequalities by ethnicity, gender identity and sexual orientation, but not by age, sex or FSM. Evidence of cross-neighbourhood variation in loneliness disparities across gender identity were found for one version of this variable, which distinguishes between males, females, gender-diverse young people and those who prefer not to say their gender (Model 3A in Appendix 1), but not for a version that distinguishes between cisgender, gender diverse and those who prefer not to say their gender (Model 3B). Loneliness was lower among Black, Asian and other ethnic groups (versus whites), higher among females, gender diverse and those who preferred not to report their gender (versus males), and higher among sexual minorities and those who preferred not to report their sexual orientation (versus heterosexual), and these inequalities vary across neighbourhoods.

Table 3 shows the associations between neighbourhood factors and loneliness in the grouped associations’ models (Models 4A and 4B) and the unique associations’ models (Models 5A and 5B). No significant associations were found for economy, work and employment, health and crime (all *P* > 0.05). Some expected associations were observed for skills deprivation among children and young people, geographical barriers, population density, perceptions of the local area and distance from home to school, whereas unexpected associations were found for adult skills deprivation and outdoor environment deprivation.

**Table 3:** Results from multilevel models with random intercepts showing associations between different types of neighbourhood factors and adolescents’ loneliness

	Grouped associations						Unique associations					
	Unadjusted			Adjusted models for year group, ethnicity, gender, sexuality and FSM			Unadjusted			Adjusted models for year group, ethnicity, gender, sexuality and FSM		
	(Model 4A)			(Model 4B)			(Model 5A)			(Model 5B)		
	Coefficient	(95% CI)	*P*	Coefficient	(95% CI)	*P*	Coefficient	(95% CI)	*P*	Coefficient	(95% CI)	*P*
*Neighbourhood level*												
Neighbourhood characteristics												
Economy, work and employment:												
Child income deprivation	0.013	(−0.015 to 0.041)	0.349	0.013	(−0.012 to 0.039)	0.298	0.001	(−0.011 to 0.013)	0.849	−0.001	(−0.012 to 0.011)	0.928
Employment deprivation	−0.014	(−0.041 to 0.014)	0.341	−0.016	(−0.041 to 0.009)	0.220	−0.002	(−0.014 to 0.011)	0.802	−0.004	(−0.016 to 0.008)	0.510
Education:												
Children and young people skills deprivation	0.065	(0.045 to 0.085)	0.000	0.030	(0.011 to 0.049)	0.002	0.007	(−0.005 to 0.020)	0.232	0.001	(−0.011 to 0.012)	0.895
Adult skills deprivation	−0.072	(−0.092 to −0.052)	0.000	−0.038	(−0.057 to −0.018)	0.000	−0.019	(−0.031 to 0.006)	0.003	−0.013	(−0.025 to 0.001)	0.030
Health:												
Health deprivation and disability	−0.001	(−0.013 to 0.011)	0.882	−0.003	(−0.014 to 0.008)	0.603	−0.001	(−0.013 to 0.011)	0.882	−0.003	(−0.014 to 0.008)	0.603
Crime:												
Crime	−0.007	(−0.019 to 0.005)	0.270	−0.002	(−0.013 to 0.010)	0.765	−0.007	(−0.019 to 0.005)	0.270	−0.002	(−0.013 to 0.010)	0.765
Barriers to housing and services:												
Geographical barriers	0.016	(0.003 to 0.030)	0.018	0.012	(−0.001 to 0.024)	0.063	0.018	(0.006 to 0.030)	0.004	0.010	(−0.001 to 0.022)	0.075
Wider barriers	−0.004	(−0.017 to 0.010)	0.584	0.004	(−0.010 to 0.017)	0.584	−0.011	(−0.023 to 0.001)	0.080	−0.001	(−0.013 to 0.011)	0.859
Living environment:												
Indoor environment deprivation	0.007	(−0.006 to 0.020)	0.310	0.009	(−0.003 to 0.021)	0.148	−0.002	(−0.015 to 0.010)	0.711	0.006	(−0.005 to 0.017)	0.290
Outdoor environment deprivation	−0.024	(−0.037 to −0.011)	0.000	−0.008	(−0.021 to 0.004)	0.193	−0.021	(−0.033 to −0.009)	0.001	−0.005	(−0.016 to 0.007)	0.412
Demographic:												
Population density	−0.027	(−0.039 to −0.014)	0.000	−0.008	(−0.020 to 0.004)	0.167	−0.027	(−0.039 to −0.014)	0.000	−0.008	(−0.020 to 0.004)	0.167
#BeeWell—local environment variables												
I feel safe in the area where I live	0.003	(−0.053 to 0.063)	0.855	0.024	(−0.036 to 0.071)	0.529	−0.173	(−0.214 to −0.131)	0.000	−0.129	(−0.167 to −0.091)	0.000
People around here support each other with their wellbeing	−0.122	(−0.190 to −0.054)	0.000	−0.073	(−0.135 to −0.011)	0.020	−0.240	(−0.283 to −0.198)	0.000	−0.180	(−0.219 to −0.141)	0.000
You can trust people around here	−0.028	(−0.093 to −0.036)	0.392	−0.066	(−0.125 to −0.007)	0.029	−0.200	(−0.239 to −0.160)	0.000	−0.167	(−0.204 to −0.131)	0.000
I could ask for help or a favour from neighbours	−0.127	(0.174 to −0.080)	0.000	−0.078	(0.122 to −0.035)	0.000	−0.220	(−0.259 to −0.180)	0.000	−0.157	(−0.193 to −0.121)	0.000
There are good places to spend your free time	−0.054	(−0.103 to −0.006)	0.028	−0.044	(−0.088 to 0.000)	0.052	−0.175	(−0.216 to −0.133)	0.000	−0.133	(−0.171 to −0.096)	0.000
*Individual level*												
Distance to school												
Distance from home to school	0.009	(−0.002 to 0.020)	0.110	0.010	(−0.000 to 0.021)	0.044	0.009	(−0.002 to 0.020)	0.110	0.010	(−0.000 to 0.021)	0.044

Statistically significant effects (*P* < 0.05) are highlighted in bold

Specifically, for Education, higher levels of skills deprivation among children and young people predicted higher loneliness in the grouped associations’ models (Models 4A and 4B)—an increase of 1 standard deviation (SD) in this index was associated with an increase of 0.065 SD in loneliness in the unadjusted model (Model 4A) and 0.030 SD in the adjusted model (Model 4B). These associations were not statistically significant in the unique associations’ models (Models 5A and 5B). Higher adult skill deprivation was associated with lower levels of loneliness in all Models 4A, 4B, 5A and 5B (respectively, *B* = −0.072, *B* = −0.038, *B* = −0.019, *B* = −0.013). In the Barriers to Housing and Services domain, higher geographical barriers were associated with significantly higher loneliness in the unadjusted Models 4A and 5A (respectively, *B* = 0.016, *B* = 0.018). Conversely, in the Living Environment domain, higher outdoor environment deprivation was associated with lower loneliness in the unadjusted Models 4A and 5A (respectively, *B* = −0.024, *B* = −0.021). In the demographic domain, higher population density was associated with lower loneliness in the unadjusted Models 4A and 5A (*B* = −0.027).

In terms of neighbourhood perceptions, higher feelings of safety were associated with lower loneliness, but only in the unique associations Models 5A and 5B (*B* = −0.173, *B* = −0.129). Higher levels of trust in local people predicted lower loneliness in Models 4B (*B* = −0.066), 5A (*B* = −0.200) and 5B (*B* = −0.167). The higher availability of good places to spend free time was associated with lower loneliness in Models 4B (*B* = −0.054), 5A (*B* = −0.175) and 5B (*B* = −0.133). Significant inverse associations with loneliness were found in all the Models 4A through 5B for neighbourhood wellbeing, support from local people (respectively, *B* = −0.122, *B* = −0.073, *B* = −0.240, *B* = −0.180), and perceived helpfulness of neighbours (respectively, *B* = −0.127, *B* = −0.078, *B* = −0.220, *B* = −0.157).

Finally, a longer distance between home and school predicted higher loneliness in the adjusted Models 4B and 5B (*B* = 0.010).

## Discussion

### Main findings of this study

Our multilevel analyses revealed that a small but statistically signification proportion of the variation in loneliness among adolescents in Greater Manchester was explained at the neighbourhood (LSOA) level. Moreover, ethnic, gender, identity and sexual orientation inequalities in loneliness varied across neighbourhoods. Neighbourhood characteristics influenced loneliness: loneliness was higher among adolescents in those neighbourhoods with higher skills deprivation among children and young people, lower skills deprivation among adults, higher geographical barriers, lower outdoor environment deprivation and lower population density, as well as in those neighbourhoods where perceptions of the local environment (feeling safe, trust in local people, feeling supported by local people, seeing neighbours as helpful and the availability of good places to spend free time) were more negative. A longer distance from home to school was also associated with higher loneliness.

### What is already known on this topic

A small, but significant, proportion of variation in adolescent loneliness is explained at the community level, as was found in previous research focusing on older adolescents (16–24-year-olds) and when using larger and more heterogeneous geographic units.^9^ We found that loneliness was highest among those from sexual minority groups, consistent with research with older adolescents^9^ and adults,^31^ and higher among females (versus males), consistent with previous research on older adolescents.^9^ Ethnic minorities reported lower loneliness than Whites, consistent with previous work in mid-to-late/adolescence,^2^^,^^9^ but in contrast to research with adults.^32^ This discrepancy highlights the need to examine loneliness from a life-course perspective, identifying changing social contexts and individual needs that contribute to experiences of loneliness at certain points in the life course, but not others.^5^ However, it is also important to consider that related factors, such as immigration background, may play a role in adolescent loneliness. For example, a study of Danish adolescents by Madsen *et al*.^33^ found that immigrants, but not descendants of immigrants, were at increased risk of loneliness among minority ethnic groups. In addition, our own research has indicated that experiencing discrimination based on race, skin colour or where you were born is associated with significantly increased risk of loneliness.^34^ What’s more, differences in loneliness between ethnic groups may reflect cultural differences in family structure and support. Research with older adults indicates that some ethnic minority groups are more likely to live in multigenerational households and have better social networks, leading to reduced loneliness^35^^,^^36^ Further research on the intersection of these and other factors, such as language, is required to advance our understanding of what are undoubtedly complex underpinning processes.

In line with earlier work with adults^37^ and older adolescents,^9^ we found that gender, ethnic and sexual orientation inequalities in loneliness differ across geographic areas. Although it could be argued that some of the gender effects on loneliness might be driven by reporting bias, that is unlikely given meta-analytic work in the field.^38^ Perceived neighbourhood characteristics, particularly social cohesion/social capital, are also important risk factors for elevated loneliness, in line with findings from previous studies focusing on adults^39^ and older adolescents.^9^

### What this study adds

This study adds to the limited existing literature on community effects on adolescent loneliness (e.g. Marquez *et al*.^9^). It is the first to focus on small neighbourhood units, and considers a younger age group than prior work. It draws on post- (as opposed to pre-) Covid-19 data and considers multiple neighbourhood characteristics spanning various domains (as opposed to perceptions of the local area only) measured at the LSOA level.

Our findings provide evidence that place and social capital are significant contributory factors for loneliness among school-aged adolescents. First, we found that living in an area where there is high education and skills deprivation among children and young people and geographical barriers (i.e. a longer distance to a post office, a GP surgery, a primary school, and a local store or supermarket) is associated with higher reporting of loneliness. Skills deprivation and geographic barriers to services are linked directly to employment prospects and the promotion of relational mobility,^40^ thus increasing friendship and social opportunities and freedom to choose who one has relationships with. Given this, it may be no surprise that these are important risk factors for youth loneliness. Such findings highlight the importance of the Levelling-Up agenda in the United Kingdom to tackle loneliness.^41^

Our findings also show that neighbourhood social cohesion, assessed in terms of safety, trust and support in neighbours, is just as important among school-aged youth in mitigating loneliness as for adults.^39^^,^^42^^,^^43^ That finding offers some suggestions for intervention, although we must be mindful that the community context, expressed by high overall social cohesion, has been shown, among adults at least, to benefit people from dominant social groups and those who can exploit local social capital (e.g. by having the resources and ability to join local groups). On the other hand, members of marginalized groups often have limited resources to take up such opportunities or are actively excluded from them.^44^^,^^45^

Social marginalization, then, needs to be considered in the planning and delivery of any intervention, given the findings in previous studies about social cohesion, but also due to the findings discussed earlier that socially marginalized groups (e.g. minority gender, or sexuality) of school-aged adolescents report more loneliness and that these inequalities vary across neighbourhoods. This means place is important for loneliness because it shapes adolescents’ experiences, but it may be more likely to induce loneliness among minority groups in some neighbourhoods more than in others, depending on the characteristics of those neighbourhoods. Also, although previous research has shown evidence of vulnerability to loneliness (varying by place) with regards to sexual orientation minorities,^9^ the present study is the first study to demonstrate that this also applies to gender minorities during adolescence. An important next step will be to investigate the characteristics of neighbourhoods where the risk of loneliness is greatest (or least) for those vulnerable groups, to understand how and why place matters. In particular, experiences of discriminations should be investigated as a moderator of the effect of neighbourhood on loneliness for those groups.

We found some counter-intuitive results—namely, negative associations between loneliness and adult education and skills deprivation and outdoor environment deprivation, respectively. The outdoor environment deprivation finding was only evident in our unadjusted models, and so may stem from socio-demographic differences that are accounted for in the adjusted models. However, the adult skills deprivation finding was not sensitive to model specification, and so we must consider the possibility that it stems from factors such as differences in family composition and availability of adults in households. Relatedly, such differences may also explain ethnicity effects. Unfortunately, while plausible, this explanation must remain speculative because the data required to empirically verify it (such as family composition information) is not available for this dataset. An alternative perspective is that the counter-intuitive effects observed reflect the fact that the metrics underpinning the adult education and skills deprivation and outdoor environment deprivation data may simply not be of immediate concern to adolescents, given they are derived from indicators including English proficiency, air pollution and road traffic accidents.

As with all research of this nature, it is important to consider the relative size of the neighbourhood effect observed. In the absence of clear guidance on what might be considered an ‘important’ or ‘practically meaningful’ neighbourhood ICC for loneliness, it is perhaps useful to contrast to loneliness ICCs in other developmental contexts such as schools (i.e. do neighbourhoods matter more or less for loneliness than other developmental contexts?), while also considering neighbourhood effects for other outcomes such as mental health and wellbeing (i.e. do neighbourhoods matter more or less for loneliness than other outcomes?). In relation to the former, the very limited amount of available evidence indicates that school differences account for between 0.6 and 2.2% of the variation in loneliness among children and adolescents.^46^ Thus, we can tentatively conclude that the influence of neighbourhoods and schools on loneliness are broadly analogous. In relation to the latter, other studies have provided evidence that the neighbourhood ICCs for internalizing symptoms and wellbeing among adolescents are comparable (≅1%) to that reported here for loneliness,^47^ but higher (≅5%) for outcomes such as externalizing problems.^48^

In sum, although our findings indicate that variation in loneliness is primarily attributable to factors other than place (e.g. differences between individuals), the small but significant effects of neighbourhoods (and their characteristics) observed here can still be considered practically meaningful given that previous research shows that the contexts in which people reside are constant, meaning that important effects can be accrued over long periods of time.^49^^,^^50^

Our findings offer some support for the idea that loneliness may be addressed at the local community level. That, of course, could be either directly (i.e. targeting loneliness and related concepts in social relationships) or indirectly (i.e. addressing inequalities, social cohesion, etc., that proved to be correlates of loneliness for the current sample). Previously, Lim *et al*.^6^ argued that delivering and implementing only one type of solution (subscribing to a one-size-fits-all approach) is both problematic and limiting. That is because loneliness is associated with a multitude of correlates and risk factors, some more modifiable (i.e. socio-environmental) than others (i.e. demographic; Lim *et al*.). Thus, interventions provided at both the individual and local community levels will be the most effective at reducing loneliness. In a recent meta-analysis of interventions for loneliness,^51^ very few studies addressed interventions for loneliness at the community level. Thus, future research will want to develop community-focused interventions for loneliness and establish their effectiveness at reducing youth loneliness.

### Limitations of this study

First, this study was cross-sectional in nature, limiting causal inference. Future longitudinal research can establish temporal precedence/separation of explanatory and response variables. Second, except for the #BeeWell local environment items, which were generated through a detailed consultation process,^52^ our neighbourhood characteristics were not necessarily attuned to the needs and preferences of adolescents. This may explain the rather limited number of noteworthy associations (and/or counter-intuitive associations) that were observed. Future consultative research should build adolescent-derived indices of neighbourhood characteristics that are most meaningful to them in terms of their loneliness. Such work could also explore how adolescents conceptualize the geographic boundaries of their neighbourhoods since, despite using the most granular geographic unit available (and hence, the one most likely to mirror adolescents’ perceptions), we were still reliant on administrative data to define ‘place’.

Third, we note that the self-reported neighbourhood characteristics (e.g. feeling safe, availability of places to spend free time) were more consistently and strongly associated with adolescent loneliness than the characteristics drawn from administrative data. This could result from same source bias,^53^ with shared-method variance inflating coefficients, and/or lonelier individuals being more negatively disposed towards their environments.^15^ However, as noted above, it could also simply be that the self-reported neighbourhood characteristics were more salient to adolescents’ wellbeing (recalling the fact that the development of the #BeeWell survey, including the selection of the local environment items, included an extensive consultation process with young people).

Fourth, this study used a single-item loneliness scale. Following scoping work and consultation with experts on existing approaches to loneliness measurement, the ONS noted that the ‘gold standard’ is to use direct measures such as the one that we used in combination with indirect ones (e.g. the three-item UCLA Loneliness Scale^54^) given that there is variation in how people understand the term ‘loneliness’ and some people might be reluctant to admit to loneliness.^55^ However, the ONS recommends the use of the direct single-item measure used in our study in cases where survey space is a major constraint (as was the case in the #BeeWell study).

## Conclusions

This study presents original insights into the influence of neighbourhoods and their characteristics on loneliness in early-to-mid adolescence. Our findings indicate that neighbourhoods account for a small but significant proportion of the variation in adolescent loneliness, with some neighbourhood characteristics (e.g. access to services and resources, social cohesion/capital) predicting loneliness at the individual level, and levels of inequality in loneliness for some groups (e.g. gender and sexual minorities) differing across neighbourhoods. However, there are challenges associated with defining place, and a need to incorporate adolescents’ views when it comes to demarcating neighbourhoods and determining what neighbourhood characteristics are most meaningful in terms of loneliness. Nonetheless, our findings are relevant to the development of public policy in various domains, including public health, urban design, education and broader social policy. Although the variation in loneliness explained by neighbourhood differences is small, it can still be considered practically meaningful given the constancy of this developmental context and the consequent accrual of effects over long periods of time.^49^ Overall, our findings suggest that compared to national, less nuanced policy approaches, local-level interventions may be more helpful to tackle loneliness and reduce inequalities.

## Supplementary Material

Appendix_1_fdad053

## Data Availability

The socio-demographic data (e.g. ethnicity) underlying this article were provided by the 10 Greater Manchester Local Authorities as part of a controller-to-controller data sharing agreement. Said agreement specified no onward sharing, meaning that these data cannot be provided. Anonymized survey data (e.g. loneliness, local environment items) underlying this article will made publicly available at the end of 2026 via the Open Science Framework or equivalent repository. Participants or their parents/carers can withdraw their data until this time and accordingly, it is not possible to share the dataset until this period has lapsed. The neighbourhood characteristic datasets underlying this article were derived from sources in the public domain.^17^^,^^18^

## Notes

**Jose Marquez**, Research Fellow

**Pamela Qualter**, Professor

**Kimberly Petersen**, Lecturer/Researcher

**Neil Humphrey**, Professor

**Louise Black**, Research Fellow
