# Supplementary material for: Neighbourhood effects on loneliness among adolescents
Source: J Public Health (Oxf). 2023 May 12;45(3):663–75. doi: 10.1093/pubmed/fdad053 (PMC10470482; doi:10.1093/pubmed/fdad053)
Supplement: Appendix_1_fdad053 [file appendix_1_fdad053.docx]

**Appendix 1**

| Table A1. Results from Multilevel Models with Random Slopes Showing Neighbourhood Random Effects in Adolescents' Loneliness for Socio-Demographic Variables (35,008 adolescents, 1,438 neighbourhoods) | | | | | | | | | | | | | | | | | | | | |
| --- | --- | --- | --- | --- | --- | --- | --- | --- | --- | --- | --- | --- | --- | --- | --- | --- | --- | --- | --- | --- |
|  | Year group random effect | |  | Ethnicity random effect | |  | Sex random effect | |  | Gender random effect (A) | |  | Gender random effect (B) | |  | Sexuality random effect | |  | FSM random effect | |
|  | (Model 3) | |  | (Model 3) | |  | (Model 3) | |  | (Model 3) | |  | (Model 3) | |  | (Model 3) | |  | (Model 3) | |
|  | b | S.E. |  | b | S.E. |  | b | S.E. |  | b | S.E. |  | b | S.E. |  | b | S.E. |  | b | S.E. |
| *Fixed:* |  |  |  |  |  |  |  |  |  |  |  |  |  |  |  |  |  |  |  |  |
| Year 10 (ref.: year 8) | 0.136*** | (0.011) |  | 0.136*** | (0.011) |  | 0.138*** | (0.011) |  | 0.136*** | (0.011) |  | 0.136*** | (0.011) |  | 0.136*** | (0.011) |  | 0.136*** | (0.011) |
| Ethnicity - Black (ref.: white) | -0.064* | (0.027) |  | -0.063* | (0.027) |  | -0.061* | (0.027) |  | -0.064* | (0.027) |  | -0.064* | (0.027) |  | -0.063* | (0.027) |  | -0.063* | (0.027) |
| Ethnicity - Asian (ref.: white) | -0.166*** | (0.015) |  | -0.165*** | (0.015) |  | -0.165*** | (0.015) |  | -0.164*** | (0.015) |  | -0.164*** | (0.016) |  | -0.163*** | (0.015) |  | -0.166*** | (0.015) |
| Ethnicity - Other (ref.: white) | -0.016 | (0.018) |  | -0.015 | (0.018) |  | -0.021 | (0.018) |  | -0.015 | (0.018) |  | -0.015 | (0.019) |  | -0.016 | (0.018) |  | -0.016 | (0.018) |
| Sex at birth - Female (ref.: male) |  |  |  |  |  |  | 0.392*** | (0.011) |  |  |  |  |  |  |  |  |  |  |  |  |
| Gender (A) - Female (ref.: male) | 0.392*** | (0.012) |  | 0.392*** | (0.012) |  |  |  |  | 0.393*** | (0.012) |  |  |  |  | 0.393*** | (0.012) |  | 0.393*** | (0.012) |
| Gender (A) - Gender diverse (ref.: male) | 0.423*** | (0.023) |  | 0.424*** | (0.023) |  |  |  |  | 0.427*** | (0.024) |  |  |  |  | 0.423*** | (0.023) |  | 0.424*** | (0.023) |
| Gender (A) - Prefer not to say (ref.: male) | 0.176*** | (0.026) |  | 0.176*** | (0.026) |  |  |  |  | 0.176*** | (0.027) |  |  |  |  | 0.176*** | (0.026) |  | 0.176*** | (0.026) |
| Gender (B) – Gender diverse (ref: cisgender) |  |  |  |  |  |  |  |  |  |  |  |  | 0.208*** | (0.027) |  |  |  |  |  |  |
| Gender (B) - Prefer not to say (ref: cisgender) |  |  |  |  |  |  |  |  |  |  |  |  | -0.044 | (0.028) |  |  |  |  |  |  |
| Sexuality - Minority sexual orientation (ref.: heterosexual) | 0.591*** | (0.017) |  | 0.590*** | (0.017) |  | 0.622*** | (0.016) |  | 0.589*** | (0.017) |  | 0.660*** | (0.017) |  | 0.591*** | (0.017) |  | 0.591*** | (0.017) |
| Sexuality - Prefer not to say  (ref.: heterosexual) | 0.256*** | (0.020) |  | 0.257*** | (0.020) |  | 0.258*** | (0.019) |  | 0.256*** | (0.020) |  | 0.308*** | (0.020) |  | 0.259*** | (0.021) |  | 0.256*** | (0.020) |
| FSM (ref.: no FSM) | 0.053*** | (0.013) |  | 0.053*** | (0.013) |  | 0.050*** | (0.013) |  | 0.053*** | (0.013) |  | 0.060*** | (0.013) |  | 0.053*** | (0.013) |  | 0.053*** | (0.013) |
| *Random:* |  |  |  |  |  |  |  |  |  |  |  |  |  |  |  |  |  |  |  |  |
|  | 0.052 | |  | 0.045 | |  | 0.051 | |  | 0.033 | |  | 0.062 | |  | 0.049 | |  | 0.056 | |
| Neighbourhood level (S.D., 95% C.I., S.E.) | (0.026 to 0.105) | |  | (0.018 to 0.114) | |  | (0.027 to 0.095) | |  | (0.006 to 0.168) | |  | (0.047 to 0.083) | |  | (0.025 to 0.098) | |  | (0.032 to 0.097) | |
|  | (0.018) | |  | (0.021) | |  | (0.016) | |  | (0.027) | |  | (0.009) | |  | (0.017) | |  | (0.016) | |
|  | 0.049 | |  |  |  |  |  |  |  |  |  |  |  |  |  |  |  |  |  |  |
| Year group random effect (S.D., 95% C.I., S.E.) | (0.010 to 0.238) | |  |  |  |  |  |  |  |  |  |  |  |  |  |  |  |  |  |  |
|  | (0.039) | |  |  |  |  |  |  |  |  |  |  |  |  |  |  |  |  |  |  |
|  |  |  |  | 0.060 | |  |  |  |  |  |  |  |  |  |  |  |  |  |  |  |
| Ethnicity random effect (S.D., 95% C.I., S.E.) |  |  |  | (0.046 to 0.079) | |  |  |  |  |  |  |  |  |  |  |  |  |  |  |  |
|  |  |  |  | (0.008) | |  |  |  |  |  |  |  |  |  |  |  |  |  |  |  |
|  |  |  |  |  |  |  | 0.000 | |  |  |  |  |  |  |  |  |  |  |  |  |
| Sex random effect (S.D., 95% C.I., S.E.) |  |  |  |  |  |  | (0.000 to 0.000) | |  |  |  |  |  |  |  |  |  |  |  |  |
|  |  |  |  |  |  |  | (0.000) | |  |  |  |  |  |  |  |  |  |  |  |  |
|  |  |  |  |  |  |  |  |  |  | 0.062 | |  |  |  |  |  |  |  |  |  |
| Gender random effect (A) (S.D., 95% C.I., S.E.) |  |  |  |  |  |  |  |  |  | (0.043 to 0.088) | |  |  |  |  |  |  |  |  |  |
|  |  |  |  |  |  |  |  |  |  | (0.011) | |  |  |  |  |  |  |  |  |  |
|  |  |  |  |  |  |  |  |  |  |  |  |  | 0.039 | |  |  |  |  |  |  |
| Gender random effect (B) (S.D., 95% C.I., S.E.) |  |  |  |  |  |  |  |  |  |  |  |  | (0.016 to 0.098) | |  |  |  |  |  |  |
|  |  |  |  |  |  |  |  |  |  |  |  |  | (0.018) | |  |  |  |  |  |  |
|  |  |  |  |  |  |  |  |  |  |  |  |  |  |  |  | 0.086 | |  |  |  |
| Sexuality random effect (S.D., 95% C.I., S.E.) |  |  |  |  |  |  |  |  |  |  |  |  |  |  |  | (0.059 to 0.0128) | |  |  |  |
|  |  |  |  |  |  |  |  |  |  |  |  |  |  |  |  | (0.017) | |  |  |  |
|  |  |  |  |  |  |  |  |  |  |  |  |  |  |  |  |  |  |  | 0.055 | |
| FSM random effect (S.D., 95% C.I., S.E.) |  |  |  |  |  |  |  |  |  |  |  |  |  |  |  |  |  |  | (0.009 to 0.326) | |
|  |  |  |  |  |  |  |  |  |  |  |  |  |  |  |  |  |  |  | (0.050) | |
|  | 0.939 | |  | 0.940 | |  | 0.938 | |  | 0.937 | |  | 0.940 | |  | 0.937 | |  | 0.939 | |
| Individual level (S.D., 95% C.I., S.E.) | (0.931 to 0.946) | |  | (0.932 to 0.948) | |  | (0.931 to 0.946) | |  | (0.929 to 0.945) | |  | (0.931 to 0.949) | |  | (0.929 to 0.945) | |  | (0.931 to 0.946) | |
|  | (0.004) | |  | (0.004) | |  | (0.004) | |  | (0.004) | |  | (0.004) | |  | (0.004) | |  | (0.004) | |
| S.D. = Standard Deviation; S.E. = Standard Error; C.I. = Confidence Interval; *** p<0.001, ** p<0.01, * p<0.05 | | | | | | | | | | | | | | | | | | | | |
